# Resonant and Non-Resonant Impurity States Related to GaAs/AlGaAs Quantum Well Sub-Bands

**DOI:** 10.3390/ma18010017

**Published:** 2024-12-24

**Authors:** Volodymyr Akimov, Viktor Tulupenko, Roman Demediuk, Anton Tiutiunnyk, Carlos A. Duque, Alvaro L. Morales, David Laroze, Miguel Eduardo Mora-Ramos

**Affiliations:** 1 Facultad de Ciencias Básicas, Universidad de Medellín, Medellín 050026, Colombia; 2 Grupo de Materia Condensada-UdeA, Instituto de Física, Facultad de Ciencias Exactas y Naturales, Universidad de Antioquia UdeA, Medellín 050010, Colombia; 3 Physics Department, Donbas State Engineering Academy, 84313 Kramatorsk, Ukraine; 4 Physics Department, Donbas State Pedagogical University, 84100 Sloviansk, Ukraine; 5 Departamento de Física, FACI, Universidad de Tarapacá, Casilla 7D, Arica 1000000, Chile; 6 Instituto de Alta Investigación, Universidad de Tarapacá, Casilla 7D, Arica 1000000, Chile; 7 Centro de Investigación en Ciencias-IICBA, Universidad Autónoma del Estado de Morelos, Cuernavaca 62209, Mexico

**Keywords:** semiconductor heterostructures, hydrogenic impurity states, expansion method

## Abstract

The energy positions and wave function shapes of the ground and excited states of impurities, including resonance states, are studied using the expansion of the impurity wave function in basis functions. The structures under study are rectangular GaAs/AlGaAs quantum wells with four different widths. In all cases, the impurity binding energy (relative to the corresponding sub-band) has a maximum at or near the center of the quantum well, decreases as the heterointerface is approached, and apparently has a limit of 0 if the impurity moves deeper into the barrier. If the impurity moves away from the center of the quantum well, then the “center of mass” of the electron charge of non-resonant impurity states follows the impurity atom, and the “center of mass” of the electron charge of the resonant impurity states moves away from it. The effect is more pronounced for the ground and first resonance states for wider quantum wells, and the shifts reach a maximum when the impurity atom is positioned near the midpoint of the path between the quantum well center and the heterointerface.

## 1. Introduction

Since the creation of the first layered semiconductor nanostructure in 1970 [1], semiconductor quantum wells (QWs) have become the basis for many optoelectronic devices, including LEDs, lasers, optical detectors, and modulators [2], which are widely used in both everyday life and science. Nowadays, such devices continue to be improved; at the same time, semiconductor QWs are being considered for use in new designs exploiting new effects [3]. Some of the more recent types of semiconductor nanostructures such as core-shell nanotubes ([4] and others) with big enough radii can also be treated mathematically as quantum wells. Although many different material pairs are used for nanostructures, including the recently popular InGaN ([5] and others), “old materials” such as Si/SiGe and AlGaAs/GaAs remain the most in-demand in industry and science [6]. In particular, GaAs/AlGaAs is considered as a typical material couple for model calculations because it has evident theoretical advantages, such as isotropic electron effective masses, and close lattice constants for the QW and barrier materials. This makes it possible to simplify calculations and obtain clear results for further analysis. The intentional introduction of impurities into a semiconductor or semiconductor heterostructure is a common method for obtaining structures with desired optical and electrical properties [7,8,9]. Doping is mainly used to alter the conductivity type and the charge carrier concentration. Delta doping can also be used to change the energy profile and, accordingly, the operating frequency of the device. Since the degree of ionization of the impurity can be regulated by the electronic temperature/external fields, this change can be tunable [10,11,12,13]. Additionally, the impurity atoms themselves also change the energy structure of semiconductors, providing complementary energy states that directly affect the optical spectra of optoelectronic devices.

The most common methods for calculating impurity states in quantum wells are variational methods based on the pioneering work of Bastard [14]. These methods are the simplest to implement, but they only allow one to obtain energy positions of impurity states, but not their wave functions (WFs). Other approaches, which use the expansion of the impurity WF by the basic wave functions [15,16], are considered more complex, but, nevertheless, allow one to find the correct impurity WF, which can then be used to calculate optical transitions involving the impurity. Impurity states can be non-resonant or completely localized if they are located below the conduction band edge (in particular, below the bottom of the ground sub-band of the quantum well), or resonant if they have energies within the continuous spectrum of sub-bands and interact with them in a manner similar to that described by Fano [17].

In our recent work [18], we reported a method for calculating the energy spectrum and wave functions of impurity states in a quantum well of arbitrary shape. It uses the mathematical approach first proposed in [15] and takes advantage of modern computing power. The method solves the problem using the effective mass approximation and is based on the expansion of the impurity wave function in terms of the eigenfunctions of the Hamiltonian associated with the quantum well but without the Coulomb term. In the present work, we study the energy positions and binding energies of 18 resonant and non-resonant impurity states, as well as the evolution of calculated wave functions of the ground and first resonant states depending on the impurity position within and near the rectangular GaAs/AlGaAs quantum wells of several different widths.

The advantages of our work in comparison to the available literature devoted to impurity states in quantum wells can be summarized as follows.

1. Unlike most such works using some version of variational techniques, we used the expansion method that allowed us to obtain not only energy positions but also the wave functions of impurity states, which is the key to many detectable impurity-related properties of the structures, including optical spectra.
2. We focus on the resonant impurity states, where the IS wave functions are superimposed on the continuous spectra of the sub-bands.
3. In comparison to the few existing works using the expansion method, we have access to contemporary computing power, which allowed us to calculate and report much bigger numbers of excited states (seventeen) than in [16] (one) or in [19] (three). Generally, a higher number of excited states requires much more refined numerical calculation. In addition, it allowed us to provide much better visualization.
4. Due to the above, we were able to find, for the first time, some patterns in the WFs reported below, generalize them to many states, and study the dynamics.

It is worth noting that our results are expected to be qualitatively relevant for any semiconductor material where effective mass approximation is applicable. For that reason, we report the calculated energies either as relative values or in terms of effective Rydbergs, so that they can be used for semiquantitative comparisons with such materials.

The abbreviations used in this work include the following: QW—quantum well; BE—impurity binding energy; WF—wave function; IS—impurity state.

## 2. Materials and Methods

**Figure 1:**
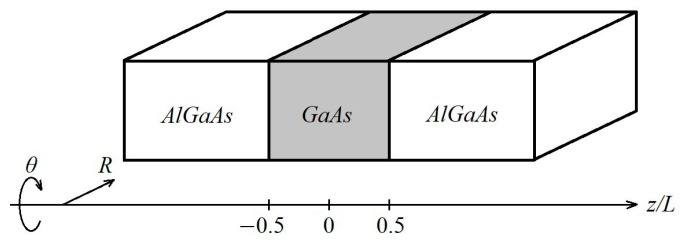
Schematics of the considered structures.

We consider layered Al_x_Ga_x−1_As/GaAs/Al_x_Ga_x−1_As heterostructures (as depicted in Figure 1) with AlGaAs layers forming potential barriers wide enough to avoid the probability of notable tunneling (assumed to be infinite for mathematical purposes). The layer of GaAs between the barriers is narrow enough to provide a notable quantum confinement. The method that we use assumes radial symmetry and uses cylindrical coordinates *R*, *ϴ,* and *z*, where *z* denotes the direction of growth of the heterostructure (perpendicular to the layers) and *R* lies in the plane of the quantum well, with *R* = 0 being the position of the impurity center and heterointerfaces at *z* = −0.5*L* and *z* = 0.5*L*, where *L* is the width of the GaAs layer or the width of the QW. The spatial position z_D_ of an impurity atom in the z-direction in our calculation varies from 0 (well center) to 0.5*L* (heterointerface) to *L* (within the barrier). Negative z_D_ values are ignored due to the symmetry.

The parameters of the materials and some other useful data are presented in Table 1. We neglected the difference in the effective mass and dielectric permittivity of the well and barrier materials. For further grounding of the parameters, see the text below. The aluminum content x in the barrier material is not specified because the only parameter it influences within our model is the well depth $V_{b}$, and there are contradictory experimental data in the literature about the connection of x and $V_{b}$. Therefore, we simply assume that the fabricator can produce a well structure with a specified well depth based on his knowledge of the specific technology. For the same reason, we do not include any presumable effect on the band offset of the thermal tensile strain reported for similar structures in [20,21].

**Figure 2:**
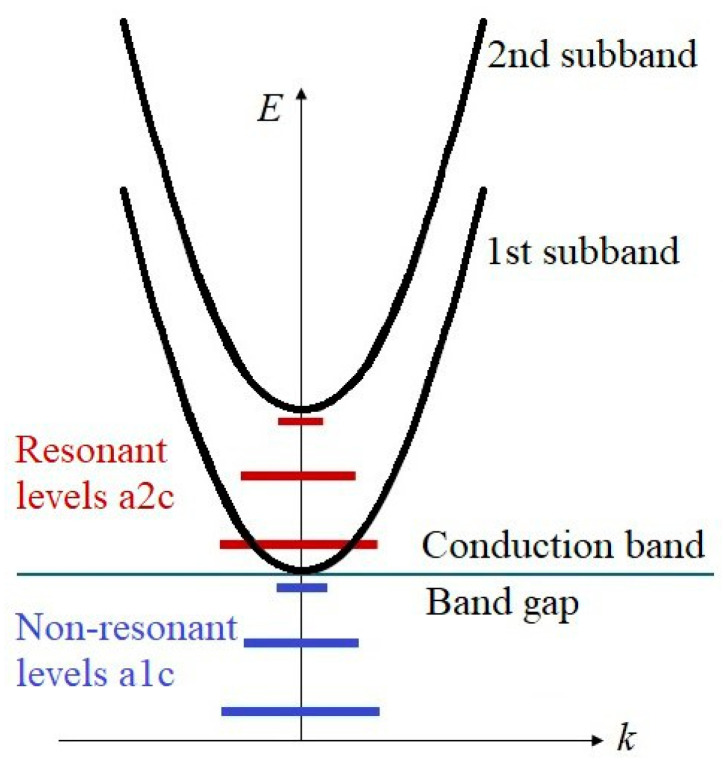
Energy structure of our QWs.

The energy structure of our quantum wells is schematically represented in Figure 2. The barriers of the well confine the movement of an electron in the z dimension, allowing energies to become quantized and form a series of sub-bands. Each sub-band is a continuous spectrum due to the free movement of the electron in two other dimensions. The distance between sub-bands depends on the well width and depth and the shape of the well. Introduction of the Coulomb potential of the impurity atom generates a series of energy levels (impurity states, IS) confined in all three dimensions to below the bottom of each sub-band, similar to a Rydberg series. If the energy distance between sub-bands is large enough, some of the ISs associated with higher sub-bands appear in the background of the continuous spectrum of lower sub-bands and are referred to as resonant. For all structures, we consider the ISs attached to the 2nd sub-band as resonant. The classification of impurity states is based on our mathematical model and is described below.

To find the impurity energy states and corresponding wave functions, we used the mathematical model first proposed in [15] and the algorithm described in detail in [18]. The impurity WFs are sought as a solution of the time-independent Schrodinger equation with a Hamiltonian consisting of a kinetic part in cylindric coordinates, a heterostructure profile *V*(*z*), and a Coulomb potential of the impurity center:

(1) $$H=-\frac{\hbar^{2}}{2m^{*}}\frac{\partial^{2}}{\partial z^{2}}-\frac{\hbar^{2}}{2m^{*}}\left(\frac{\partial^{2}}{\partial R^{2}}+\frac{1}{R}\frac{\partial }{\partial R}+\frac{1}{R^{2}}\frac{\partial^{2}}{\partial \theta^{2}}\right)+V\left(z\right)\;-\frac{e^{2}}{4\pi \varepsilon \varepsilon_{0}\sqrt{R^{2}+{\left(z-z_{D}\right)}^{2}}}.$$

Zero energy corresponds to the well bottom for the rectangular QW, $V\left(z\right)=\left\{\begin{array}{c} V_{b},\left|z\right|>L/2\\ 0,\;\left|z\right|\leq L/2 \end{array}\right\}$, *e* is the unit charge.

The solution is presented as

(2) $$\Psi \left(R,\theta ,z\right)=\exp\left(ia\theta \right)\sum_{j}f_{j}\left(R\right)\xi_{j}\left(z\right),$$

where *θ* is the azimuthal angle, *a* is the azimuthal quantum number, *i* is the imaginary unit, $f_{j}\left(R\right)$ are some expansion coefficients, which are calculated numerically as described in [18], and $\xi_{j}\left(z\right)$ are eigenfunctions of the Hamiltonian *H*_0_, similar to Equation (1) but without the Coulomb term and the radial part:

(3) $$H_{0}=-\frac{\hbar^{2}}{2m^{*}}\frac{\partial^{2}}{\partial z^{2}}+V\left(z\right).$$

$\xi_{j}\left(z\right)$ correspond to one-dimensional wave functions of the QW sub-bands. The eigenstates of *H*_0_ are the energies of the sub-bands *E_j_*.

The classification of calculated impurity energies *E_abc_* and wave functions *Ψ_abc_* follows [16]. *a*, *b,* and *c* are three quantum numbers where *a* is presented in Equation (2) and is denoted by the letter “s” for *a* = 0, “p” for *a* = 1, and “d” for *a* = 2. The second quantum number *b* refers to the QW sub-band to which the impurity state is attached to and is counted from 1, which means that *E*_1_ is the QW’s ground sub-band energy and $\xi_{1}\left(z\right)$ is the QW’s ground sub-band wave function. The third quantum number *c* starts from 1 and describes a series of states below the *b*-th sub-band for a given *a* in order of increasing energy. For example, according to the classification above, s11 is the ground impurity state, *E*_s11_ is the ground impurity energy, and *Ψ*_s11_ is the corresponding wave function.

The assumptions and limitations of this method are the ones inherent in the band theory, such as effective mass approximation and hydrogenic impurity with the point Coulomb center model. In addition, important numerical parameters are the number of expansion terms (7 for all cases in our work) and integration (finite differences) limits, which are discussed in [18]. In this work, we have performed the necessary convergence checks and can state that all presented results are stable with respect to numerical parameters, within an error of no more than 1%.

As stated, we chose GaAs/AlGaAs QWs, bearing in mind their advantages as mentioned in the Introduction. The depth of GaAs quantum wells is 55 Ry, as stated in the paper of Stopa and DasSarma [19]. Where possible, we compared our results with theirs and can state that the agreement is excellent, noting that we have many more results. It is supposed that Si atoms are used as donors for both the QW and the barriers. The disadvantage of GaAs-based structures in our case is that due to the small impurity binding energy (about 6 meV in bulk), the energies we consider in this work are also very small, on the scale several meV, so that the corresponding effects are difficult to verify experimentally. However, we expect that some fundamental results of this work are qualitatively applicable to other semiconductor materials with bigger impurity binding energies (such as Si/Si_1−*x*_Ge*_x_*).

We took the well widths L = 30, 40, 50, and 60 nm, which in terms of the Bohr radius is approximately 3, 4, 5, and 6 *r_B_*. This choice is due to the following. 1. Several values of width make it easier to trace the dependence of the binding energy and the wave function of the impurity on its position relative to the QW and on the width of the QW. 2. All QWs with the indicated depth (55 Ry) have 7 or more energy levels (sub-bands) of spatial quantization, which (as already mentioned) is sufficient for good convergence of the obtained results and which we used in our method. Narrow wells with *L* ≤ 20 nm contain less than 7 sub-bands. For such QWs, one should either reduce the number of expansion terms, and, accordingly, the numerical precision, or use big box approximation for a greater number of expansion terms, which also introduces additional sources of numerical error. In both cases, the calculations will be somewhat inconsistent with those for wider QWs. 3. The energy gaps between the lowest of these levels (which are of interest to us) belong to the terahertz range, which is a “hot” topic now. On the other hand, for the wells of *L* = 70 nm or more, the energy difference between the two lowest sub-bands is too small (0.552 Ry or less, which, in the case of GaAs/AlGaAs, is less than 4 meV). It means that the associated physical phenomena, such as spectral properties, become harder to detect and distinguish. In addition, for wider QWs, the lower impurity state attached to the second sub-band s21 for some impurity positions drops below the first sub-band and becomes non-resonant, while starting to mix with higher s1n energy states at the same time. This means that the calculation results become less stable with respect to numerical parameters and the overall picture becomes too complex to analyze. 4. In addition, such comparatively wide QWs make it possible to ignore non-parabolicity phenomena, which begin to play a role in QWs with *L* < 3*r_B_* [23]. The same can be said about the failure to consider the difference in permittivity and effective masses for quantum well and barrier materials [24]. 5. Finally, the results obtained with these QW widths can be “tried on” for materials with smaller *r_B_* values and, accordingly, for narrower QWs. In this regard, we once again highlight that one of the goals of this work is to clarify all the features of the resonant ISs using the example of the GaAs/AlGaAs QW.

Here, we present the results for the energy positions of the impurity states in units of effective Rydberg 1Ry = $\frac{\hbar^{2}}{2m^{*}r_{B}^{2}}$, where the effective Bohr radius $r_{B}=\frac{4\pi \varepsilon \varepsilon_{0}\hbar^{2}}{m^{*}e^{2}}$, lengths in the *z* direction are in units of well width *L*, and lengths in the *R* directions are in nanometers. The *z*-position of the “center of mass” of the squared magnitudes of WFs was calculated as

(4) $$z_{cm}=\int_{V}{\left|\Psi \right|}^{2}zdV=2\pi \int_{z=-\infty }^{\infty }zdz\int_{R=0}^{\infty }{\Psi (R,z)}^{2}RdR,$$

assuming that WF Ψ is normalized as usual

(5) $$\int_{V}{\left|\Psi \right|}^{2}dV=2\pi \int_{z=-\infty }^{\infty }dz\int_{R=0}^{\infty }{\Psi (R,z)}^{2}RdR=1.$$

## 3. Results and Discussion

All calculations are performed for 18 impurity states with different combinations of quantum numbers (see the description of our “*abc*” in the previous section, “Mathematical Model”). We calculated all combinations of the first three values of the quantum number “*a*” (*a* = “s”, “p”, “d”), the first two values of the quantum number “*b*” (*b* = 1, 2), where *b* = 1 corresponds to the first (ground) sub-band and *b* = 2 corresponds to the second sub-band, and the first three values of the quantum number “*c*” (*c* = 1, 2, 3), which number the excited states. In all our studied quantum wells, the states with *b* = 1 on the energy scale are below the first sub-band of spatial quantization (Ea1c < E1), and the states with *b* = 2 lie between the first and second sub-bands of the QW (*E*_1_ < *E_a_*_2*c*_ < *E*_2_). This means that the *a*1*c* states are non-resonant, and the *a*2*c* states are resonant. Resonant ISs exist above the gap against the background of a continuous energy spectrum of lower QW sub-band(s). We define the binding energy of IS *Ɛ_abc_* as the difference between the respective sub-band energy *E_b_* and the energy of IS *E_abc_*: *Ɛ_abc_* = *E_b_* − *E_abc_*.

**Figure 3:**
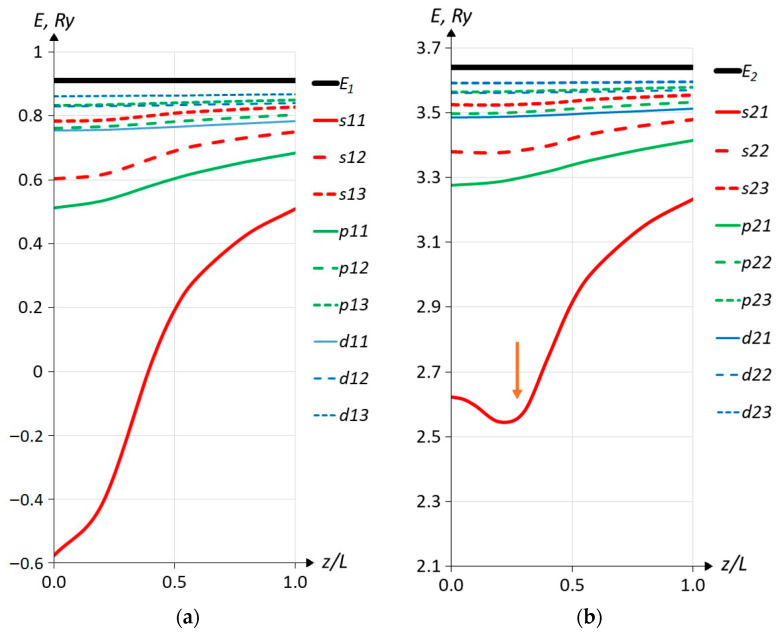
Energies of impurity levels depending on the impurity position for non-resonant (**a**) and resonant (**b**) impurity states. The heterointerface corresponds to *z*/*L* = 0.5. The quantum well width is *L* = 30 nm. *E*_1_ and *E*_2_ are the energies of the bottom of the first and second sub-bands, respectively. The red arrow in the right panel indicates the position of the maximum (one of two) of the squared modulus of the wave function for the second level of spatial quantization.

Figure 3 shows the energies of all 18 ISs as a function of the position of the impurity atom in a 30 nm wide QW in units of the well width. The zero energy corresponds to the bottom of the QW (see the term *V*(*z*) in Equation (1)). The zero-coordinate *z* is at the center of the well, and *z* = 0.5 corresponds to the right heterointerface between the well and the barrier. The QW is symmetric about its center *z* = 0, so we only show results for positive *z*. Non-resonant states are presented on the left side of the figure, and, accordingly, resonant states are on the right side of the figure. Solid black horizontal lines mark the levels (sub-band bottoms) of spatial quantization. In both panels, the energy scale and its zero are the same. First of all, attention is drawn to the fact that the extrema of binding energies (by impurity binding energy, BE, in this work, we mean the energy distance between the impurity state and the bottom of the related sub-band) of non-resonant and resonant (at least the first few resonant) impurity states are located at different points along the z-axis inside the quantum well. This becomes clear if we remember that the WF of the IS by its nature is a sub-band WF perturbed by the Coulomb potential. The perturbation is that the impurity WF is a superposition of the WFs of the dimensional quantization levels, and the main contribution is given by the WF of the level with which the IS is associated. It is therefore not surprising that the maximum binding energy is located at or near the position of the maximum of the squared modulus of the WF for the corresponding spatial quantization level. This is exactly so for a non-resonant IS. Indeed, both the squared modulus of the wave function for the first spatial quantization level and the maximum BE are at the same point, namely at *z* = 0. The positions of the maximum (one of the two) of the squared WF for the second level and the maximum BE for the “ground” resonant state s21 are not in the same position, but are near each other, with the maximum BE slightly shifted toward the center of the QW. In Figure 3b, the red arrow indicates the maximum of the WF for the second spatially quantized energy level. Obviously, this reflects the fact that the second-largest contribution to the resonant impurity WF comes from the WF of the first spatial quantization level. To our knowledge, similar behavior for the s21 impurity state depending on the impurity position in the QW was observed only in [25], in which only this resonant state was calculated using the variational method, and impurities in the barriers were not considered (the impurity was located only inside the QW). The second feature that we can note is that the alternation of resonant states practically repeats the behavior of localized ISs.

**Figure 4:**
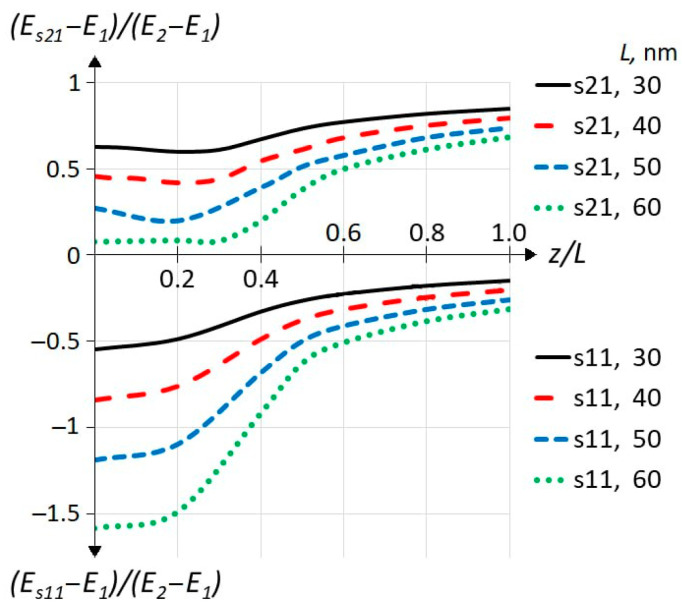
Relative energies of impurity levels for impurity states s11 (non-resonant, lower part of the figure) and s21 (resonant, upper part of the figure) depending on the impurity position. The heterointerface corresponds to *z*/*L* = 0.5. The widths of quantum wells are *L* = 30, 40, 50, and 60 nm.

Figure 4 shows the energies of the s11 and s21 states as a function of the impurity position for different QW widths. Here, unlike in Figure 3, the relative units of energy are presented on the ordinate axis. The point is that for different well widths, all energy levels (including dimensional quantization levels) will have different energies. Therefore, to show the same behavior of impurity energy states depending on the position for all well widths studied, we use here the relative energy units according to (*E − E*_1_)/(*E*_2_ *− E*_1_). Thus, the zero value of the ordinate axis corresponds to the position of the first level (sub-band) of spatial quantization *E*_1_, and 1 corresponds to the position of the second level (sub-band) of spatial quantization *E*_2_ in this figure. We see that the curves have the same form as for the 30 nm QW. However, it should be borne in mind that the difference between *E*_1_ and *E*_2_ decreases with increasing QW width as follows: 2.73, 1.60, 1.05, and 0.74 Ry for L = 30, 40, 50, and 60 nm. This means that the absolute values of the binding energies do not increase with increasing QW width (as one might mistakenly conclude from Figure 4), but rather decreases somewhat. This is clearly seen in Table 2, which presents the binding energies for all calculated ISs and well widths for the case of an impurity in the QW center *z_D_* = 0. The order of states in decreasing energy for each sub-band follows the sequence presented in [18] for central doping, “s-p-s-(p,d)-s-(p,d)”, which resembles the sequence of energy for Rydberg’s atom. For example, for a 30 nm QW, we see the following order of arrangement of impurity energy levels under the first sub-band: s11(1.487), p11(0.398), s12(0.308), d11(0.157), p12(0.150), and s13(0.128). Here, in brackets, the corresponding energy values presented in Table 2 are shown. However, we do not see any reliable parallel to the 1/n^2^ energy rule for a hydrogenic atom, which would be valid for any well width. Further analysis of Table 2 allows us to make the following statements, which are true for all the data presented. (i) BE for each IS decreases monotonically with the width of the QW. (ii) The BE of the non-resonant state is greater than the BE of the corresponding resonant state; in relative terms, this is more pronounced for ISs with a larger BE. (iii) In terms of relative BE change, ISs with a bigger BE are less sensitive to the width of the QW; as for resonant ISs *E_a_*_2*c*_, this means that they are more sensitive than corresponding non-resonant ISs *E_a_*_1*c*_. Numerically, in all statements, we speak of a relative effect from 50% to 2%. Our findings are consistent with the results obtained in the literature for sufficiently wide quantum wells for both variation [25,26] and non-variational [16,19] methods. In the limit of an infinitely wide QW, the BEs should have values equal to the corresponding BEs of a hydrogen-like atom with a fundamental energy of 1 Ry. The mathematical model we use is generally not applicable to this limit, but the calculated BEs of the s11 ground state clearly appear to approach 1 effective Rydberg, which is the theoretical energy of a hydrogen impurity in a bulk semiconductor.

Table 3 presents numerical data on the change in BE for all studied values of the well width and impurity states for an impurity in the heterointerface (*z_D_* = 0.5*L*) and inside the barrier (*z_D_* = *L*). The BE of the corresponding state in the well center is taken as 100%, and the cells of the table indicate how much it decreases for the specified positions.

The largest relative decrease in BE always occurs in the ground state s11 (50–60% for *z* = 0.5*L* and 70–80% for *z* = 1.0*L*), and the second largest relative change occurs in the first resonant state s21 (28–33% for *z* = 0.5*L* and 60–66% for *z* = 1.0*L*). A general but not absolute rule is that larger BEs have a larger relative change. A larger well width always increases the relative change in BE, but not by much.

**Figure 5:**
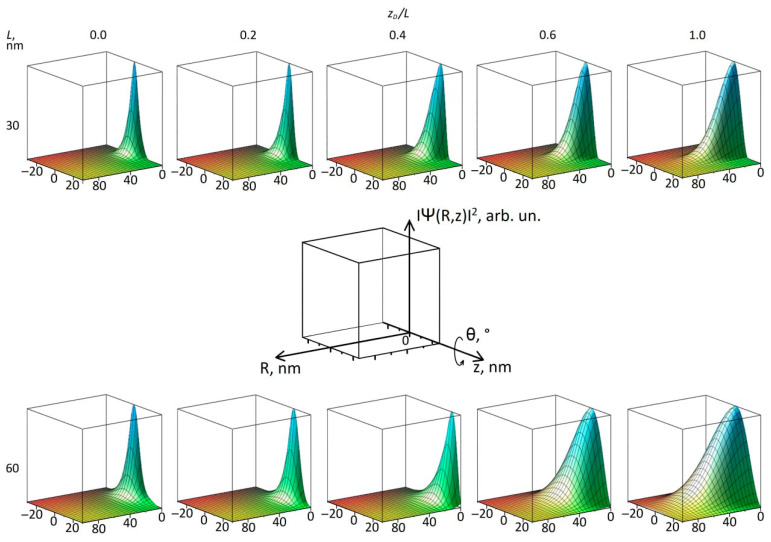
*R–z* plots for the cross-sections of the squares of wave functions for the s11 (non-resonant) impurity states for different impurity positions *z_D_*; QW width *L* = 30 nm (upper part), and *L* = 60 nm (lower part of the figure).

Now let us pay attention to the impurity wave function. A rule of thumb to keep in mind when interpreting impurity energies in terms of impurity WFs is that a larger BE corresponds to a smaller average distance between the positive point charge of an impurity center and the negative charge of an electron (the square of the WF) of the impurity state. Our heterostructures always have azimuthal symmetry with respect to R, but they are symmetric with respect to z only when the impurity is located at the center of the well *z_D_* = 0. In this case, the decrease in BE means that the square of the WF is stretched in space (and, accordingly, its amplitude is somewhat reduced, since the square of the WF modulus is normalized to 1) and its “center of mass” along the *z*-axis remains in the same position. When the impurity is moved away from the center, the symmetry is broken. Asymmetry means that the “center of mass” (or center of negative charge, which in our case is the same thing) of the WF of the specific IS is also shifted from the center of the QW, but in a different way than the impurity atom. However, before we move on to the discussion, some clarification is needed regarding Figure 5 (and the similar figure below). As can be seen from Equation (2), the modulus of the impurity WF does not depend on the azimuthal angle *θ*, since the square of the exponential factor is always equal to 1. This means that we have two dimensions of interest on which our WFs depend, *R* and *z*, and so to graphically represent the distribution of the electron charge, we use three-dimensional graphics, in which the squares of the amplitudes of the wave functions are plotted against the *R*–*z* coordinates, as shown in Figure 5. Here, the cross-sections of the squares of the modulus of the impurity WFs of the ground (non-resonant) state s11 are plotted for QW widths *L* = 30 and 60 nm for different positions of the impurity *z_D_* relative to the well center. In this Figure and in the similar figure below, we use the same orientation in our *R*–*z* plots, so that the *z*-axis corresponds to the bottom right edge of the coordinate box, and the *R*-axis is in the middle of the bottom face of the coordinate box and points from right to left. The impurity displaces in a positive *z* direction. The entire square of the WF module can be imagined by rotating the graph around the *z*-axis. To more clearly show the propagation of the WF in the *R* direction, all surfaces have different conventional units for the WF, although they are all normalized in 3D to 1. In *z*, WFs are well-localized between the barriers of the heterostructure (−15..15 nm for *L* = 30 nm and −30..30 for *L* = 60 nm). For the WFs near the center of the QW, the dimensions of the WF are quite comparable. When the impurity moves towards the barrier, the WF remains inside the well, the interaction between the negative and positive charges weakens, the BE decreases (see Figure 3 and Figure 4), and the WF spreads into *R*. In the case of a wide well, the WFs inside the well appear “thicker”, indicating its lower binding energy. Indeed, for a wider well, the WF is less confined, and this is reflected in the decrease in BE in Table 3. For its position in the barrier, the WF has a significantly larger propagation in *R*, which further decreases the BE, but the relative effect (Table 3) is rather small due to the lower BE in the well center.

**Figure 6:**
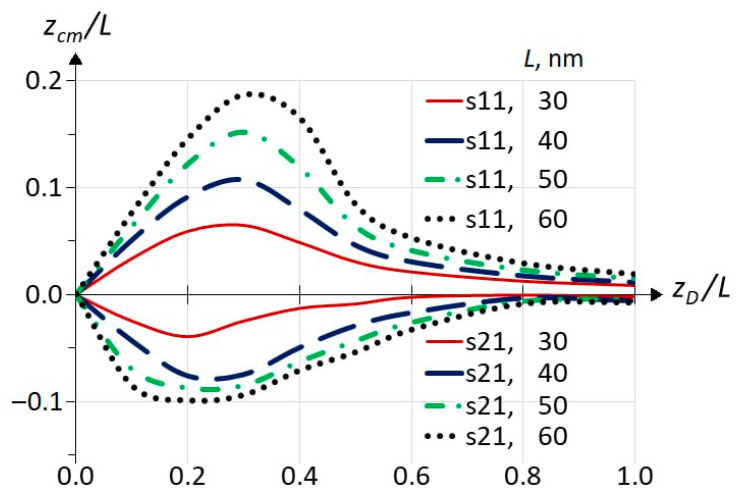
Positions of center of mass (*z_cm_*) of the WFs of the s11 (**upper panel**) and s21 (**lower panel**) impurity states for different quantum well widths depending on the impurity position. The heterointerface corresponds to *z* = 0.5.

In Figure 6, one can see the center of mass (calculated by Equation (4)) of the IS WF as a function of the impurity position for the ground s11 (upper panel) and first resonant s21 (lower panel) ISs for different well widths. The first and the most interesting observation is that the center of mass of the s11 WF displaces away from the well center towards the impurity atom, while the s21 WF displaces away from the well center in the opposite direction. It can be stated that the WFs of all calculated ISs behave qualitatively in the same way: all non-resonant WFs *a*1*c* follow impurity, and all resonant *a*2*c* move away from it. In the case of excited states, this effect is much weaker than for s11 and s21 ISs. We did not perform a full study for the *a*3*c* states (those below the third sub-band) but a few selected points have a positive displacement as in the case of *a*1*c* states. Below, we will discuss Figure 6 separately for the top and bottom panels, starting with the top.

**Figure 7:**
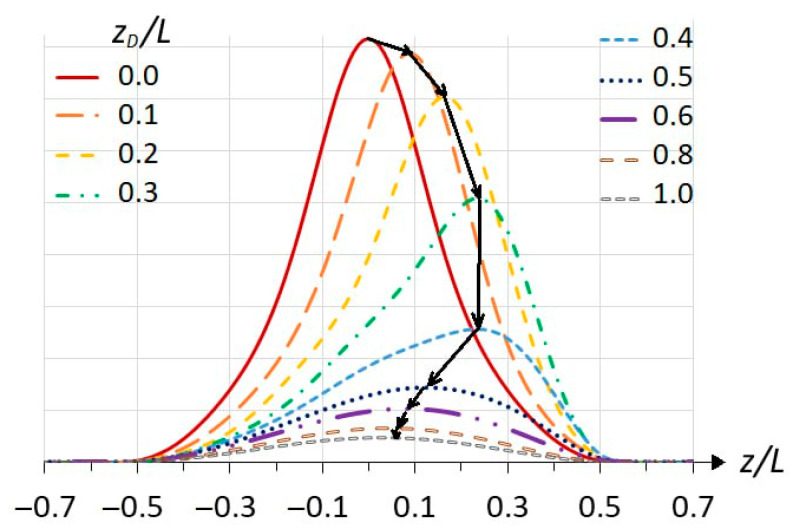
Cross-sections of the normalized squares of the wave functions of the impurity state s11 at R = 0 for different impurity positions indicated in the inset. The quantum well width L = 30 nm. The black arrows show the sequence of the curves in order of increasing impurity displacement from the quantum well center.

In Figure 5, one can see the displacement of the square of the s11 state WF relative to the center of the QW as the peak approaches the observer; however, this is better seen in Figure 7. It shows the cross-sections of the squares of the s11 wave functions at *R* = 0 for different positions of the impurities indicated in the inset, which correspond to the images on the right faces of the upper cubes in Figure 5. All WFs are normalized in 3D, so a smaller average value of one curve relative to another means that the first WF is more stretched out in the R dimension. The behavior of the s11 WF is due to the attraction of the positive charge of the atom and the repulsion by the barriers, but in any case, the WF remains locked between the barriers. This means that at small displacements of the impurity from the well center, the WF (electron charge) follows the positively charged atom—its attraction prevails, although we see the appearance of charge separation, or an electric dipole moment. The maximum asymmetry of the WF relative to the well center is within *z_D_* = 0.2…0.4*L*. With further removal of the impurity, the repulsion by the barrier prevails, and the influence of the Coulomb potential on the shape of the *z*-profile decreases. In this case, the charge separation increases, and the WF is stretched in *R* (Figure 5), so that the amplitude of the curves in Figure 7 decreases, and the WF restores symmetry relative to the QW center. In Figure 6, this corresponds to the right-hand side, where the curves tend to zero.

**Figure 8:**
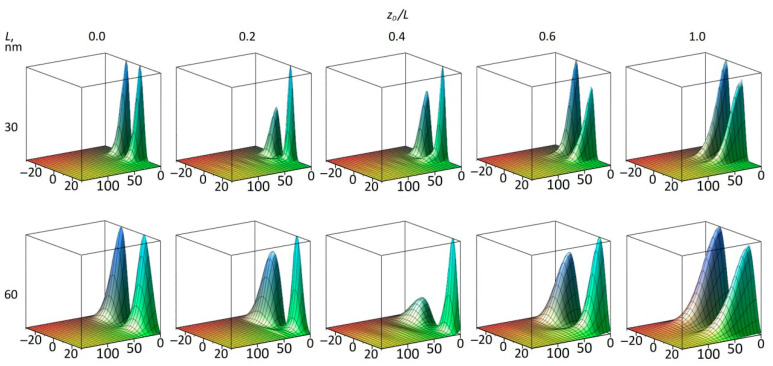
*R–z* plots for the cross-sections of the squares of wave functions for the s21 (resonant) impurity states for different impurity positions *z_D_*; QW width *L* = 30 nm—upper part, and *L* = 60 nm—lower part of the figure.

**Figure 9:**
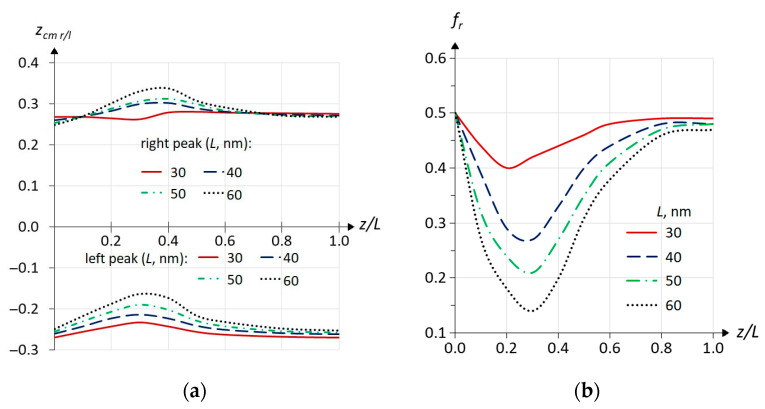
(**a**) Positions of the center of mass for the squares of the wave function amplitudes for s21 impurity states (resonant): right peak—upper panel, left peak—lower panel; (**b**) relative amplitude of the left peak depending on the impurity position. The heterointerface corresponds to z= 0.5. The width of the quantum wells is indicated on the graphs.

We now turn to a discussion of the s21 IS. Unlike s11, when the symmetry is broken and the impurity is shifted to the right, the center of mass of the s21 state shifts to the left of the well center (Figure 6). Figure 8 shows the squares of the s21 wave functions on the *R*–*z* plane in the same way that Figure 5 shows the squares of the s11 WF. As we discussed in [18], in *z,* the WF qualitatively reproduces the WF of the second sub-band, which is antisymmetric with respect to the QW center, and the square of the WF has two peaks on the *R*–*z* plot. The waves that are barely visible on the left side of the graphs are not the result of numerical errors, but a reflection of the resonant nature of the s21 state. They arise due to the interaction of the impurity state with the continuum of the first sub-band. Their discussion is beyond the scope of this article. In Figure 8, it can be seen that the breaking of the symmetry leads to the change in the shape of the WF peaks. To illustrate the change in amplitude for each peak and the influence of the first sub-band, the squares of the wave functions are shown in different scales for each cube. At *z* = 0, the peaks are expectedly symmetrical. When the impurity moves to the right, the right peak (having a Coulomb potential funnel nearby) becomes sharper with a larger maximum, and the left peak becomes blunter but wider. With further movement of the impurity and its appearance in the barrier (*z_D_* > 0.5), the WF tends to restore symmetry under stretching in *R*, as in the case of the s11 state. Another point to note is that for both QW widths, at some point (between 0.4 and 0.6 for the narrow well, and between 0.6 and 1.0 for the wide well), the peak maxima invert, and the right peak becomes slightly lower than the left. Now, the question arises whether the center of mass of the IS WF s21 shifts to the left (Figure 6) due to the different shift of the peaks or due to the redistribution of the mass between the peaks (the redistribution of the probability density of finding an electron in an impurity state along the z-axis). In principle, such behavior can also be explained like the explanation of the behavior of the ${\left|\Psi_{s11}\right|}^{2\;}$, namely because of competition between the attraction of ${\left|\Psi_{s21}\right|}^{2\;}$ to the positive charge of the atom and its repulsion by the barrier to which the impurity atom moves. However, the complicating factor here is the presence of two peaks, and so we present the following estimates below. The position of the IS WF’s center of mass (of electron charge) can be expressed in terms of the mass fractions $f_{r(l)}$ and center of mass positions $z_{cm\;r(l)}$ for the right-hand and left-hand peaks, respectively, as $z_{cm\;}=f_{\;r}z_{cm\;r}+f_{\;l}z_{cm\;l}$, assuming $f_{\;r}+f_{\;l}=1$. Keeping in mind that since the WF of the second sub-band is antisymmetric and the IS WF tends to qualitatively follow its shape, one can easily distinguish the two peaks numerically since their WFs have different signs. If we ensure that the WF for the right peak is negative and that it is positive for the left peak, for the normalized IS WF, we can calculate $f_{l,r}$ and $z_{cm\;l,r}$ as follows:

$$f_{l,r}=\int_{V}h(\pm \Psi )\times {\left|\Psi \right|}^{2}dV$$

$$z_{cm\;l,r}=\frac{1}{f_{l,r}}\int_{V}h(\pm \Psi )\times {\left|\Psi \right|}^{2}zdV,$$

where *h*() is the Heaviside step function. This estimation is not perfect as it is influenced by the waves on the WFs due to the interaction with the sub-band continuum (Figure 6), and the two reasons above cannot be separated easily. Moreover, the results can be modified by using an increasingly larger *R* for numerical integration. Here, it is necessary to keep in mind that due to the cylindrical coordinate system, visually, small waves at larger R values have a larger contribution to the integrals. However, we have verified that this contribution is relatively small. The results of the calculations are presented in Figure 9. The figure shows that both peaks are mostly shifted in the positive direction (to the right, where the impurity atom moves), with the left peak decreasing in amplitude. However, this peak is wider and overall contains most of the WF (most of the probability for finding the electron charge), so the total center of mass is shifted away from the impurity center to the left.

## 4. Conclusions

We calculated and analyzed the energy positions (binding energies) and wave functions of 18 impurity states (nine non-resonant and nine resonant) in rectangular GaAs/AlGaAs quantum wells of four different widths depending on the impurity position. The calculations were performed using the expansion method in the effective mass approximation (hydrogen-like impurity). The binding energies of all states have a maximum at or near the well center, decrease significantly near the barriers, and tend to zero deep in the barriers. These results are consistent with those available in the literature.

As expected, the most prominent results are those of the ground impurity state and the first resonance state (the first below the second sub-band of the quantum well). The other impurity states have significantly lower binding energies and much greater spatial localization of their wave functions in the QW plane. Qualitatively, they have similar behaviors depending on the change in the impurity position, but are much less pronounced in both absolute and relative values. Wider QWs, as a rule, provide larger changes in relative values, but smaller ones in absolute values.

For an impurity at the center of a quantum well, its wave functions are symmetric for non-resonant states and antisymmetric for resonant impurity states. The displacement of an impurity atom from the center of the quantum well breaks the symmetry relative to the center of the quantum well, with the greatest asymmetry observed for the position of the impurity approximately halfway from the center to the barrier. The effect of such asymmetry is different for resonant and non-resonant impurity states: the former are shifted towards the impurity, and the latter are shifted away from the impurity. For an impurity inside the barrier, its wave functions remain trapped in the quantum well and regain symmetry about the center of the quantum well with their increased propagation in the plane of the quantum well. This is because these WFs are inherently sub-band WFs perturbed by the Coulomb potential. Therefore, they always remain inside the well, even if the impurity center is located deep inside the barrier. For an impurity far from the heterointerface, the perturbation is reduced and our ISs become indistinguishable from sub-band states.

The impurities in semiconductor nanostructures have a wide range of effects and applications (see [9] and references therein). Below, we outline some qualitative conclusions and mention observed effects and unsolved problems, which can be addressed numerically in the future using the materials of this article as a reference point.

Impurity states affect the charge carrier mobility by changing the density of states and causing impurity scattering, which can be dominant in quantum wells even in the case of background (technological) impurity [27]. The impurity state wave functions are necessary to numerically calculate the scattering cross-section; otherwise, it can be estimated using the localization degree obtainable from our figures. For more rigorous results, WFs can also be used to calculate the probability of final electron states in k-space for Monte Carlo simulation.

Another notable point of interest is the so-called intracenter inversion mechanism, as well as a possible laser based on it [28,29,30] or other proposed semiconductor devices [10,11,31] involving intracenter optical transitions. The energy differences between the ground and excited impurity states give us the working frequencies of such devices. For s21–s11 transitions of our considered structures and conditions, they correspond to 3.2 Ry (19 meV or 4.6 THz) and lower, and wavelengths of 65 μm and bigger, which means that they are in the terahertz region (the selection rules for dipole matrix elements allow transitions between states of different parities, so this transition is preferable). WFs are needed to obtain dipole matrix elements for oscillator strengths and then, based on the Fermi golden rule and theoretical or empirical spectral line broadenings, the intracenter addition to absorption and emission spectra can be predicted numerically. Qualitatively, the effect of the opposite spatial displacement of the a1c and a2c WFs, which we report in this article, seems unfavorable to a2c–a1c intracenter transitions because it should reduce their overlap integral; however, as soon as the displacement occurs within the well borders, it is not expected to be prohibiting, and precise calculations may reveal some favorable parameter combinations, especially if we add a transverse electric field as one of the parameters.

One more expected effect of spatial displacements of impurity wave functions in cases of impurities displaced from the center is the variation in the electric dipole moment of the well with temperature, as electrons occupy higher impurity states at higher temperatures, changing their mean position in space. However, the effect is expected to be minor unless the impurity concentration is so high that our one-particle approximation is no longer valid. The correct simulation of such an effect with higher impurity concentrations should include autoconsistent simulations with the addition of Hartree potential due to charge redistribution to the Hamiltonian of the problem (Equation (4)).

In addition, the resonant impurity-state WFs are key to the study of Fano resonances [17,32] observed in various semiconductor structures, including quantum wells and superlattices [33,34,35], specifically regarding the asymmetry of the spectral peaks associated with such states. Unlike the impurity WF in the gap, which can be found for a specific energy only, the resonant WFs can be calculated using our method for any energy within the continuum of the QW sub-band. Here, we present the WFs for the energies corresponding to the maximum localization of the WF. However, the rigorous reproduction of the spectral shape will require the study of the evolution of the WF in the immediate vicinity of this energy. We expect the asymmetry of such an evolution, which can illustrate and allow numerical reproduction of the observed asymmetry of the spectral line shapes in QWs.

Additionally, this article can be used as a reference point for a more general fundamental problem of electronic states of a hydrogenic atom confined in one dimension. All energies are given in effective Rydbergs so that they can be easily compared to the results of similar structures made of other materials.

Summarizing the above, we believe that the added value of this article compared to other papers devoted to impurities in (and near) a quantum well is the following. (i) To our knowledge, there is a larger number of calculated excited impurity states, including resonant ones. (ii) We obtained and visualized impurity wave functions. (iii) We found and described regularities, particularly the displacement of the WFs in the z-axis for non-symmetric configurations. (iv) Our findings have semiquantitative applicability to any semiconductor material, allowing effective mass approximation.

## Figures and Tables

**Table 1:** Parameters of the material and calculations.

Parameter	Value
Effective mass m* (*m*_0_)	0.067 [22]
Relative dielectric permittivity *ε*	12.4 [22]
Effective Rydberg energy unit (meV)	5.80
$\mathrm{Conduction}\;\mathrm{band}\;\mathrm{edge}\;\mathrm{discontinuity}\;V_{b}$ (well depth) (Ry)	55
Well width L (nm)	30..60
Impurity position z_D_ (L)	0..1
Number of expansion members	7

**Table 2:** Binding energy (Ry) for different impurity states (*abc*) and quantum well widths for an impurity located at the center of the quantum well.

*L*, nm	s11	s12	s13	s21	s22	s23
30	1.487	0.308	0.128	1.016	0.260	0.114
40	1.347	0.296	0.125	0.869	0.242	0.109
50	1.249	0.287	0.123	0.765	0.228	0.105
60	1.177	0.280	0.121	0.686	0.215	0.101
	p11	p12	p13	p21	p22	p23
30	0.398	0.150	0.078	0.362	0.142	0.075
40	0.382	0.147	0.077	0.339	0.137	0.073
50	0.368	0.144	0.076	0.320	0.132	0.072
60	0.355	0.141	0.075	0.301	0.128	0.069
	d11	d12	d13	d21	d22	d23
30	0.157	0.081	0.049	0.154	0.079	0.048
40	0.156	0.080	0.048	0.151	0.078	0.047
50	0.155	0.080	0.048	0.148	0.078	0.047
60	0.153	0.079	0.048	0.145	0.076	0.046

**Table 3:** Relative decreases in the binding energy $e_{abc}\left(z\right)=\left(\frac{E_{abc}\left(0\right)-E_{abc}\left(z\right)}{E_{abc}\left(0\right)}\right)\times 100\%$ for an impurity located at the heterointerface e_abc_ (*z_D_* = 0.5) and at a distance of one well width from the center of the quantum well *e_abc_* (*z_D_* = 1.0) compared to the binding energy at the well center.

	$e_{abc}\left(z=0.5\right),\%;$						$e_{abc}\left(z=1.0\right),\%$					
*L*, nm	s11		s12		s13		s21		s22		s23	
30	51.7	73.0	28.3	47.7	19.5	34.4	28.7	60.1	15.8	38.1	9.7	26.4
40	55.4	76.1	32.1	52.4	22.4	39.2	28.9	62.6	17.4	41.8	11.0	30.3
50	58.1	78.3	34.9	55.8	24.5	42.4	33.4	64.4	17.6	44.4	11.5	32.5
60	60.3	80.1	37.2	59.0	26.5	45.6	32.6	65.7	19.5	46.5	10.9	34.6
	p11		p12		p13		p21		p22		p23	
30	23.1	43.2	14.0	28.7	10.3	21.8	17.1	37.9	10.6	24.7	6.7	18.8
40	27.7	48.9	17.7	34.0	13.0	26.0	20.1	42.8	12.4	29.2	8.2	22.0
50	31.6	53.3	20.2	37.6	14.6	29.1	22.2	46.3	13.7	31.9	11.2	25.2
60	34.4	56.7	22.1	41.3	16.1	32.2	25.6	49.2	14.8	35.1	10.1	27.5
	d11		d12		d13		d21		d22		d23	
30	7.0	18.5	4.9	13.6	4.1	12.3	6.5	17.6	3.8	11.5	4.2	10.5
40	10.9	25.0	7.5	17.5	6.2	14.6	9.3	23.2	5.1	15.4	4.3	14.9
50	13.6	30.4	8.8	21.4	6.3	18.9	10.8	27.1	7.7	20.6	6.4	17.2
60	16.4	34.1	11.5	25.5	10.5	23.1	13.1	31.0	9.2	22.3	8.7	21.7

## Data Availability

The original contributions presented in this study are included in the article. Further inquiries can be directed to the corresponding authors.

## References

[1] Esaki L., Tsu R. (1970). Superlattice and Negative Differential Conductivity in Semiconductors. IBM J. Res. Dev..

[2] Fox M., Ispasoiu R., Kasap S., Capper P. (2017). Quantum Wells, Superlattices, and Band-Gap Engineering. Springer Handbook of Electronic and Photonic Materials.

[3] Dubowski J.J., Sugioka K. (2020). Bandgap Engineering of Quantum Semiconductor Microstructures. Handbook of Laser Micro- and Nano-Engineering.

[4] Prete P., Wolf D., Marzo F., Lovergine N. (2019). Nanoscale spectroscopic imaging of GaAs-AlGaAs quantum well tube nanowires: Correlating luminescence with nanowire size and inner multishell structure. Nanophotonics.

[5] Zhou S., Wan Z., Lei Y., Tang B., Tao G., Du P., Zhao X. (2022). InGaN quantum well with gradually varying indium content for high-efficiency GaN-based green light-emitting diodes. Opt. Lett..

[6] Ning C.Z., Dou L., Yang P. (2017). Bandgap engineering in semiconductor alloy nanomaterials with widely tunable compositions. Nat. Rev. Mater..

[7] McCluskey M.D., Haller E.E. (2021). Dopants and Defects in Semiconductors.

[8] Weidner M., Fuchs A., Bayer T.J., Rachut K., Schnell P., Deyu G.K., Klein A. (2019). Defect Modulation Doping. Adv. Funct. Mater..

[9] Holmberg V.C., Helps J.R., Mkhoyan K.A., Norris J.D. (2013). Imaging Impurities in Semiconductor Nanostructures. Chem. Mater..

[10] Tulupenko V., Duque C.A., Demedyuk R., Belichenko Y., Duque C.M., Akimov V., Poroshin V., Fomina O. (2012). On the possibility of tuning the energy separation between space-quantized levels in a quantum well. Philos. Mag. Lett..

[11] Duque C.A., Akimov V., Demediuk R., Belykh V., Tiutiunnyk A., Morales A.L., Restrepo R.L., Nalivayko O., Fomina O., Mora-Ramos M.E. (2015). About possible THz modulator on the base of delta-doped QWs. Superlattices Microstruct..

[12] Tulupenko V., Duque C.A., Morales A.L., Tiutiunnyk A., Demediuk R., Dmytrychenko T., Fomina O., Akimov V., Restrepo R.L., Mora-Ramos M.E. (2016). Background impurities in Si0.8 Ge0.2/Si/Si0.8 Ge0.2 n-type delta-doped QW. Phys. Status Solidi (b).

[13] Akimov V., Tulupenko V., Duque C.A., Morales A.L., Demediuk R., Tiutiunnyk A., Laroze D., Kovalov V., Sushchenko D. (2021). Background impurities and a delta-doped QW. Part II: Edge doping. Semicond. Sci. Technol..

[14] Bastard G. (1981). Hydrogenic impurity states in a quantum well: A simple model. Phys. Rev. B.

[15] Vinter B. (1982). Influence of charged impurities on Si inversion-layer electrons. Phys. Rev. B.

[16] Blom A., Odnoblyudov M.A., Yassievich I.N., Chao K.-A. (2003). Donor states in modulation-doped Si/SiGe heterostructures. Phys. Rev. B.

[17] Fano U. (1961). Effects of Configuration Interaction on Intensities and Phase Shifts. Phys. Rev..

[18] Akimov V., Tulupenko V., Demediuk R., Tiutiunnyk A., Duque C.A., Morales A.L., Laroze D., Mora-Ramos M.E. (2024). Numerical proceeding to calculate impurity states in 2D semiconductor heterostructures. Res. Sq..

[19] Stopa M., DasSarma S. (1989). Calculated shallow-donor-level binding energies in GaAs-AlxGa_1−x_As quantum wells. Phys. Rev. B.

[20] Prete P., Cingolani R., Rinaldi R., Ploog K.H. (1994). Thermal strain effects on the excitonic states in GaAs/AlxGa_1−x_As multiple quantum wells. J. Appl. Phys..

[21] Chen G., Sun G., Ding Y.J., Prete P., Miccoli I., Lovergine N., Shtrikman H., Kung P., Livneh T., Spanier J.E. (2013). Direct Measurement of Band Edge Discontinuity in Individual Core–Shell Nanowires by Photocurrent Spectroscopy. Nano Lett..

[22] Poerschke R., Madelung O. (1991). Semiconductors. Group IV Elements and III-V Compounds.

[23] Chaudhuri S., Bajaji K.K. (1984). Effect of nonparabolicity on the energy levels of hydrogenic donors in GaAs-Ga1-xAlxAs quantum well structures. Phys. Rev. B.

[24] Fraizzoli S., Bassani F., Buczko R. (1990). Shallow donor impurities in GaAs-Ga1-xAlxAs quantum well structures: Role of the dielectric-constant mismatch. Phys. Rev. B.

[25] Yen S.T. (2002). Theory of resonant states of hydrogenic impurities in quantum wells. Phys. Rev. B.

[26] Greene R.L., Bajaj K.K. (1983). Energy levels of hydrogenic impurity states in GaAs-Ga1−xAlxAs quantum well structures. Solid State Commun..

[27] Tschirky T., Mueller S., Lehner C.A., Fält S., Ihn T., Ensslin K., Wegscheider W. (2017). Scattering mechanisms of highest-mobility InAs/Al*x*Ga1−*x*Sb quantum wells. Phys. Rev. B.

[28] Pavlov S.G., Hübers H.W., Böttger U., Zhukavin R.K., Shastin V.N., Hovenier J.N., Redlich B., Abrosimov N.V., Riemann H. (2008). Terahertz Raman laser based on silicon doped with phosphorus. Appl. Phys. Lett..

[29] Kagan M.S., Yassievich I.N. (2002). Resonant states and THz lasing in SiGe quantum well structures δ-doped with boron. Physica E.

[30] Altukhov I.V., Chirkova E.G., Sinis V.P., Kagan M.S., Gousev Y.P., Thomas S.G., Wang K.L., Odnoblyudov M.A., Yassievich I.N. (2001). Towards Si1ÀxGex quantum-well resonant-state terahertz laser. Appl. Phys. Lett..

[31] Tsyplenkov V.V., Shastin V.N. (2018). On the Intracenter Relaxation of Shallow Arsenic Donors in Stressed Germanium. Population Inversion under Optical Excitation. Semiconductors.

[32] Limonov M.F., Rybin M.V., Poddubny A.N., Kivshar Y.S. (2017). Fano resonances in photonics. Nat. Photonics.

[33] Holfeld C.P., Löser F., Sudzius M., Leo K., Whittaker D.M., Köhler K. (1998). Fano Resonances in Semiconductor Superlattices. Phys. Rev. Lett..

[34] Kibis O.V., Kolodny S.A., Iorsh I.V. (2021). Fano resonances in optical spectra of semiconductor quantum wells dressed by circularly polarized light. Opt. Lett..

[35] Fan P., Yu Z., Fan S., Brongersma M.L. (2014). Optical Fano resonance of an individual semiconductor nanostructure. Nat. Mater..

